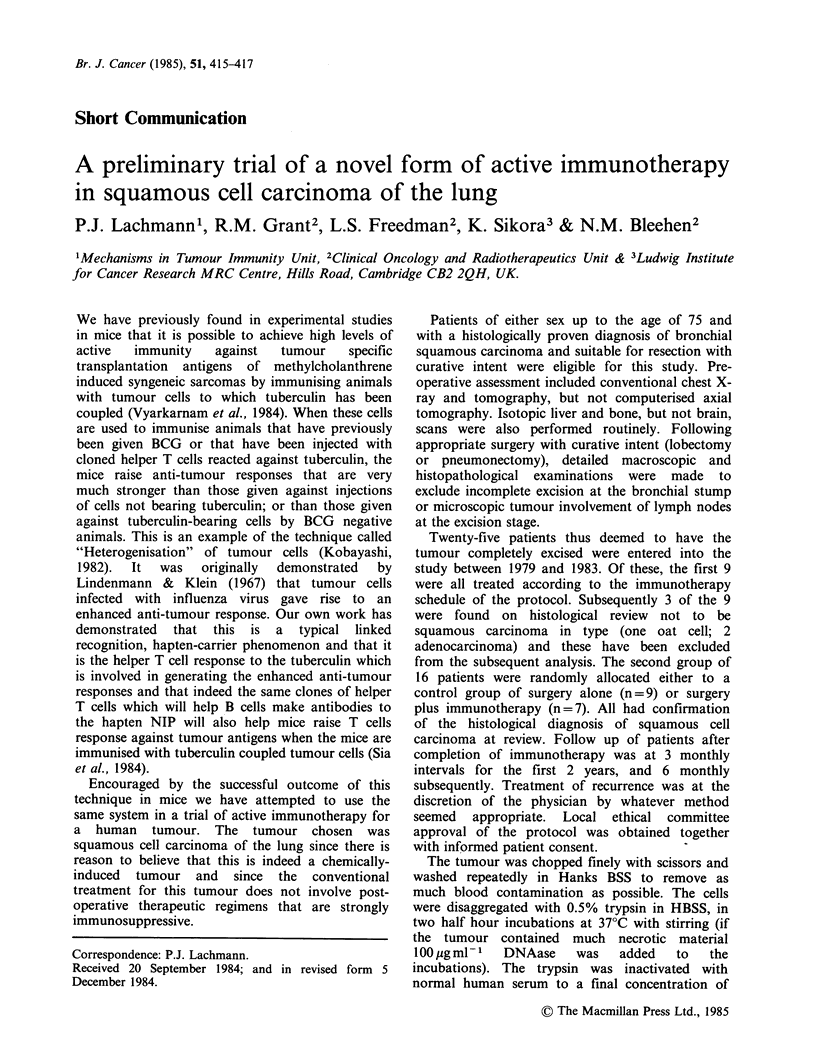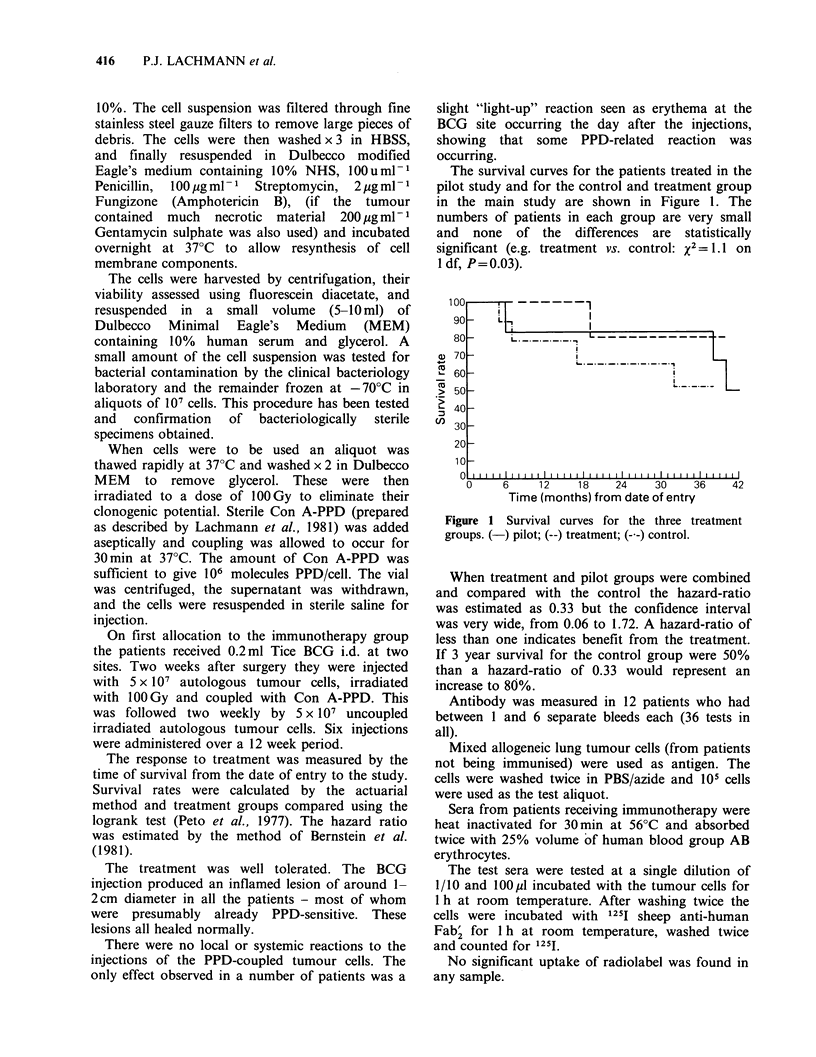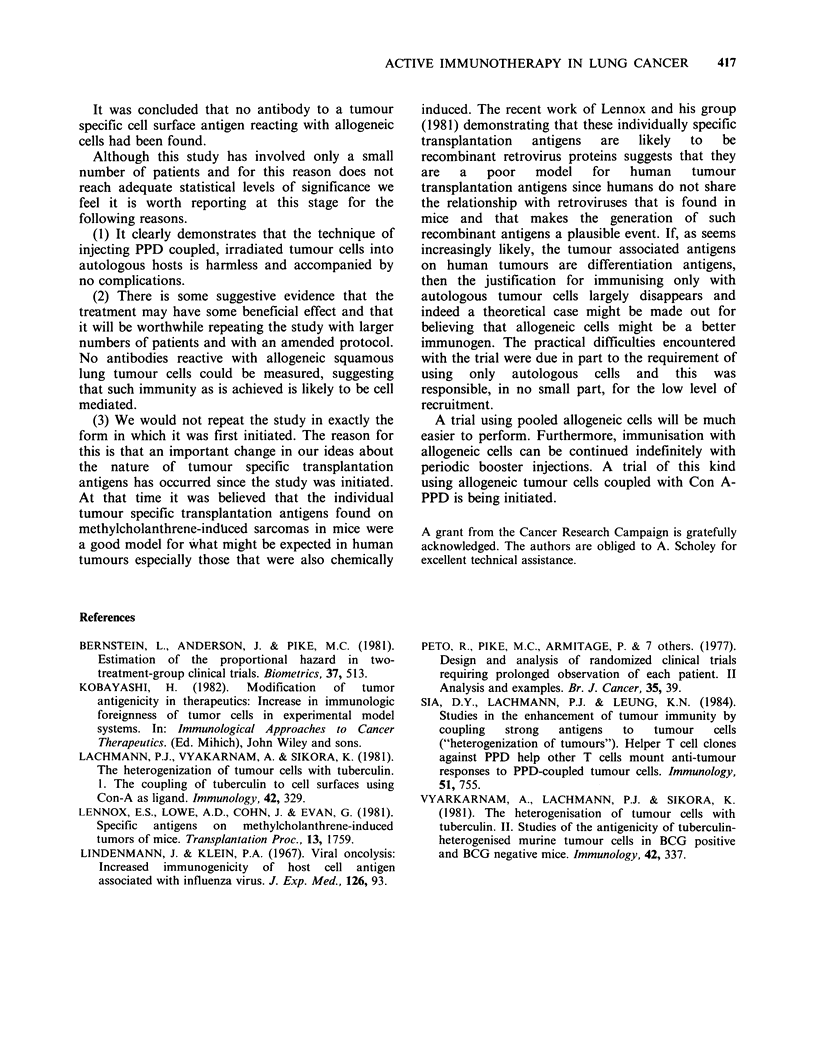# A preliminary trial of a novel form of active immunotherapy in squamous cell carcinoma of the lung.

**DOI:** 10.1038/bjc.1985.56

**Published:** 1985-03

**Authors:** P. J. Lachmann, R. M. Grant, L. S. Freedman, K. Sikora, N. M. Bleehen


Br. J. Cancer (1985), 51, 415–417

### Short Communication

### A preliminary trial of a novel form of active immunotherapy in squamous cell carcinoma of the lung

P.J. Lachmann[1], R.M. Grant[2], L.S. Freedman[2], K. Sikora[3] & N.M. Bleehen[2]

[1]*Mechanisms in Tumour Immunity Unit,* [2]*Clinical Oncology and Radiotherapeutics Unit &* [3]*Ludwig Institute for Cancer Research MRC Centre, Hills Road, Cambridge CB2 2QH, UK.*

We have previously found in experimental studies in mice that it is possible to achieve high levels of active immunity against tumour specific transplantation antigens of methylcholanthrene induced syngeneic sarcomas by immunising animals with tumour cells to which tuberculin has been coupled (Vyarkarnam *et al.*, 1984). When these cells are used to immunise animals that have previously been given BCG or that have been injected with cloned helper T cells reacted against tuberculin, the mice raise anti-tumour responses that are very much stronger than those given against injections of cells not bearing tuberculin; or than those given against tuberculin-bearing cells by BCG negative animals. This is an example of the technique called "Heterogenisation" of tumour cells (Kobayashi, 1982). It was originally demonstrated by Lindenmann & Klein (1967) that tumour cells infected with influenza virus gave rise to an enhanced anti-tumour response. Our own work has demonstrated that this is a typical linked recognition, hapten-carrier phenomenon and that it is the helper T cell response to the tuberculin which is involved in generating the enhanced anti-tumour responses and that indeed the same clones of helper T cells which will help B cells make antibodies to the hapten NIP will also help mice raise T cells response against tumour antigens when the mice are immunised with tuberculin coupled tumour cells (Sia *et al.*, 1984).

Encouraged by the successful outcome of this technique in mice we have attempted to use the same system in a trial of active immunotherapy for a human tumour. The tumour chosen was squamous cell carcinoma of the lung since there is reason to believe that this is indeed a chemically-induced tumour and since the conventional treatment for this tumour does not involve post-operative therapeutic regimens that are strongly immunosuppressive.

Correspondence: P.J. Lachmann.
Received 20 September 1984; and in revised form 5 December 1984.

Patients of either sex up to the age of 75 and with a histologically proven diagnosis of bronchial squamous carcinoma and suitable for resection with curative intent were eligible for this study. Pre-operative assessment included conventional chest X-ray and tomography, but not computerised axial tomography. Isotopic liver and bone, but not brain, scans were also performed routinely. Following appropriate surgery with curative intent (lobectomy or pneumonectomy), detailed macroscopic and histopathological examinations were made to exclude incomplete excision at the bronchial stump or microscopic tumour involvement of lymph nodes at the excision stage.

Twenty-five patients thus deemed to have the tumour completely excised were entered into the study between 1979 and 1983. Of these, the first 9 were all treated according to the immunotherapy schedule of the protocol. Subsequently 3 of the 9 were found on histological review not to be squamous carcinoma in type (one oat cell; 2 adenocarcinoma) and these have been excluded from the subsequent analysis. The second group of 16 patients were randomly allocated either to a control group of surgery alone ($n=9$) or surgery plus immunotherapy ($n=7$). All had confirmation of the histological diagnosis of squamous cell carcinoma at review. Follow up of patients after completion of immunotherapy was at 3 monthly intervals for the first 2 years, and 6 monthly subsequently. Treatment of recurrence was at the discretion of the physician by whatever method seemed appropriate. Local ethical committee approval of the protocol was obtained together with informed patient consent.

The tumour was chopped finely with scissors and washed repeatedly in Hanks BSS to remove as much blood contamination as possible. The cells were disaggregated with 0.5% trypsin in HBSS, in two half hour incubations at 37°C with stirring (if the tumour contained much necrotic material $100\,\mu g\,ml^{-1}$ DNAase was added to the incubations). The trypsin was inactivated with normal human serum to a final concentration of

© The Macmillan Press Ltd., 1985

10%. The cell suspension was filtered through fine stainless steel gauze filters to remove large pieces of debris. The cells were then washed ×3 in HBSS, and finally resuspended in Dulbecco modified Eagle's medium containing 10% NHS, $100\,u\,ml^{-1}$ Penicillin, $100\,\mu g\,ml^{-1}$ Streptomycin, $2\,\mu g\,ml^{-1}$ Fungizone (Amphotericin B), (if the tumour contained much necrotic material $200\,\mu g\,ml^{-1}$ Gentamycin sulphate was also used) and incubated overnight at 37°C to allow resynthesis of cell membrane components.

The cells were harvested by centrifugation, their viability assessed using fluorescein diacetate, and resuspended in a small volume (5–10 ml) of Dulbecco Minimal Eagle's Medium (MEM) containing 10% human serum and glycerol. A small amount of the cell suspension was tested for bacterial contamination by the clinical bacteriology laboratory and the remainder frozen at −70°C in aliquots of $10^7$ cells. This procedure has been tested and confirmation of bacteriologically sterile specimens obtained.

When cells were to be used an aliquot was thawed rapidly at 37°C and washed ×2 in Dulbecco MEM to remove glycerol. These were then irradiated to a dose of 100 Gy to eliminate their clonogenic potential. Sterile Con A-PPD (prepared as described by Lachmann *et al.*, 1981) was added aseptically and coupling was allowed to occur for 30 min at 37°C. The amount of Con A-PPD was sufficient to give $10^6$ molecules PPD/cell. The vial was centrifuged, the supernatant was withdrawn, and the cells were resuspended in sterile saline for injection.

On first allocation to the immunotherapy group the patients received 0.2 ml Tice BCG i.d. at two sites. Two weeks after surgery they were injected with $5\times10^7$ autologous tumour cells, irradiated with 100 Gy and coupled with Con A-PPD. This was followed two weekly by $5\times10^7$ uncoupled irradiated autologous tumour cells. Six injections were administered over a 12 week period.

The response to treatment was measured by the time of survival from the date of entry to the study. Survival rates were calculated by the actuarial method and treatment groups compared using the logrank test (Peto *et al.*, 1977). The hazard ratio was estimated by the method of Bernstein *et al.* (1981).

The treatment was well tolerated. The BCG injection produced an inflamed lesion of around 1–2 cm diameter in all the patients – most of whom were presumably already PPD-sensitive. These lesions all healed normally.

There were no local or systemic reactions to the injections of the PPD-coupled tumour cells. The only effect observed in a number of patients was a slight "light-up" reaction seen as erythema at the BCG site occurring the day after the injections, showing that some PPD-related reaction was occurring.

The survival curves for the patients treated in the pilot study and for the control and treatment group in the main study are shown in Figure 1. The numbers of patients in each group are very small and none of the differences are statistically significant (e.g. treatment *vs.* control: $\chi^2=1.1$ on 1 df, $P=0.03$).

**Figure 1** Survival curves for the three treatment groups. (—) pilot; (--) treatment; (-·-) control.

When treatment and pilot groups were combined and compared with the control the hazard-ratio was estimated as 0.33 but the confidence interval was very wide, from 0.06 to 1.72. A hazard-ratio of less than one indicates benefit from the treatment. If 3 year survival for the control group were 50% than a hazard-ratio of 0.33 would represent an increase to 80%.

Antibody was measured in 12 patients who had between 1 and 6 separate bleeds each (36 tests in all).

Mixed allogeneic lung tumour cells (from patients not being immunised) were used as antigen. The cells were washed twice in PBS/azide and $10^5$ cells were used as the test aliquot.

Sera from patients receiving immunotherapy were heat inactivated for 30 min at 56°C and absorbed twice with 25% volume of human blood group AB erythrocytes.

The test sera were tested at a single dilution of 1/10 and $100\,\mu l$ incubated with the tumour cells for 1 h at room temperature. After washing twice the cells were incubated with $^{125}I$ sheep anti-human $Fab'_2$ for 1 h at room temperature, washed twice and counted for $^{125}I$.

No significant uptake of radiolabel was found in any sample.

It was concluded that no antibody to a tumour specific cell surface antigen reacting with allogeneic cells had been found.

Although this study has involved only a small number of patients and for this reason does not reach adequate statistical levels of significance we feel it is worth reporting at this stage for the following reasons.

(1) It clearly demonstrates that the technique of injecting PPD coupled, irradiated tumour cells into autologous hosts is harmless and accompanied by no complications.

(2) There is some suggestive evidence that the treatment may have some beneficial effect and that it will be worthwhile repeating the study with larger numbers of patients and with an amended protocol. No antibodies reactive with allogeneic squamous lung tumour cells could be measured, suggesting that such immunity as is achieved is likely to be cell mediated.

(3) We would not repeat the study in exactly the form in which it was first initiated. The reason for this is that an important change in our ideas about the nature of tumour specific transplantation antigens has occurred since the study was initiated. At that time it was believed that the individual tumour specific transplantation antigens found on methylcholanthrene-induced sarcomas in mice were a good model for what might be expected in human tumours especially those that were also chemically induced. The recent work of Lennox and his group (1981) demonstrating that these individually specific transplantation antigens are likely to be recombinant retrovirus proteins suggests that they are a poor model for human tumour transplantation antigens since humans do not share the relationship with retroviruses that is found in mice and that makes the generation of such recombinant antigens a plausible event. If, as seems increasingly likely, the tumour associated antigens on human tumours are differentiation antigens, then the justification for immunising only with autologous tumour cells largely disappears and indeed a theoretical case might be made out for believing that allogeneic cells might be a better immunogen. The practical difficulties encountered with the trial were due in part to the requirement of using only autologous cells and this was responsible, in no small part, for the low level of recruitment.

A trial using pooled allogeneic cells will be much easier to perform. Furthermore, immunisation with allogeneic cells can be continued indefinitely with periodic booster injections. A trial of this kind using allogeneic tumour cells coupled with Con A-PPD is being initiated.

A grant from the Cancer Research Campaign is gratefully acknowledged. The authors are obliged to A. Scholey for excellent technical assistance.

**References**

BERNSTEIN, L., ANDERSON, J. & PIKE, M.C. (1981). Estimation of the proportional hazard in two-treatment-group clinical trials. *Biometrics*, **37**, 513.

KOBAYASHI, H. (1982). Modification of tumor antigenicity in therapeutics: Increase in immunologic foreignness of tumor cells in experimental model systems. In: *Immunological Approaches to Cancer Therapeutics*. (Ed. Mihich), John Wiley and sons.

LACHMANN, P.J., VYAKARNAM, A. & SIKORA, K. (1981). The heterogenization of tumour cells with tuberculin. 1. The coupling of tuberculin to cell surfaces using Con-A as ligand. *Immunology*, **42**, 329.

LENNOX, E.S., LOWE, A.D., COHN, J. & EVAN, G. (1981). Specific antigens on methylcholanthrene-induced tumors of mice. *Transplantation Proc.*, **13**, 1759.

LINDENMANN, J. & KLEIN, P.A. (1967). Viral oncolysis: Increased immunogenicity of host cell antigen associated with influenza virus. *J. Exp. Med.*, **126**, 93.

PETO, R., PIKE, M.C., ARMITAGE, P. & 7 others. (1977). Design and analysis of randomized clinical trials requiring prolonged observation of each patient. II Analysis and examples. *Br. J. Cancer*, **35**, 39.

SIA, D.Y., LACHMANN, P.J. & LEUNG, K.N. (1984). Studies in the enhancement of tumour immunity by coupling strong antigens to tumour cells ("heterogenization of tumours"). Helper T cell clones against PPD help other T cells mount anti-tumour responses to PPD-coupled tumour cells. *Immunology*, **51**, 755.

VYARKARNAM, A., LACHMANN, P.J. & SIKORA, K. (1981). The heterogenisation of tumour cells with tuberculin. II. Studies of the antigenicity of tuberculin-heterogenised murine tumour cells in BCG positive and BCG negative mice. *Immunology*, **42**, 337.